# Brain Activation and Functional Connectivity of Reappraisal and Acceptance for Anxious Events

**DOI:** 10.1523/ENEURO.0033-23.2023

**Published:** 2023-06-01

**Authors:** Masayuki Tsujimoto, Yutaka Matsuzaki, Noriki Yamaya, Ryuta Kawashima

**Affiliations:** 1 Department of Functional Brain Imaging, Institute of Development, Aging, and Cancer, Tohoku University, Sendai, Japan 980-8575; 2 Graduate School of Medicine, Tohoku University, Sendai, Japan 980-8575

**Keywords:** acceptance, anxiety, emotion regulation, functional connectivity, neural basis, reappraisal

## Abstract

Despite the significant health consequences of anxiety, the neural basis of regulation for personal anxious events is not well understood. We examined brain activity and functional connectivity during cognitive emotion regulation strategies (reappraisal and acceptance) for personal anxious events. Functional MRI (fMRI) data were obtained while 35 college students were thinking about (the control condition), reappraising, or accepting their own anxiety-provoking situations. Although reappraisal and acceptance decreased anxiety, no statistically significant differences were observed in the brain activation levels between cognitive emotion regulation strategies and the control condition. However, acceptance decreased activation in the posterior cingulate cortex and precuneus more than reappraisal. Moreover, functional connectivity with the amygdala and ventral anterior insula distinguished the emotion regulation strategies for anxiety. Reappraisal showed stronger negative functional connectivity with the amygdala and cognitive control regions than other strategies. In addition, reappraisal had negative functional coupling between the ventral anterior insula and temporal pole compared with acceptance. In contrast, acceptance showed stronger positive functional coupling between the ventral anterior insula and precentral and postcentral gyrus compared with the control condition. Our findings contribute to the understanding of emotion regulation processes by revealing the brain activity and functional connectivity patterns in reappraisal and acceptance for personal anxious events.

## Significance Statement

This study is the first to reveal the differences in brain activity and functional connectivity between reappraisal and acceptance for individual anxious events. We found that reappraisal strengthened the negative functional coupling between the cognitive control areas and emotion-processing regions. In contrast, acceptance was characterized by a reduction in the self-reflection process and strengthened the functional coupling between emotion-processing regions and self-reflective and emotion recognition areas. These results contribute to a better understanding of emotion regulation processes by providing the differences in relationships of activation in self-reflection, cognitive control, and emotion-processing regions between reappraisal and acceptance for personal anxious events. These findings may help discover interventions for mitigating the negative effects of anxiety.

## Introduction

Regulating anxiety is crucial because anxiety decreases psychological, physical, and work functioning (Kubzansky and Kawachi, 2000; Haller et al., 2014; Moran, 2016; Vancampfort et al., 2017). Understanding the processes of effective strategies for one’s anxiety helps to develop interventions aimed at reducing anxiety and understand the dysfunction of anxiety regulation. Therefore, this functional MRI (fMRI) study examined the neural basis of emotion regulation strategies for anxiety induced by the participants’ personal events.

Garnefski et al. (2001) have identified five adaptive cognitive emotion regulation strategies, and especially reappraisal and acceptance are negatively related to psychopathology (Aldao et al., 2010). Reappraisal consists of reframing the self-relevant meaning of the emotion-provoking situation (Gross and John, 2003; Etkin et al., 2015), while acceptance involves experiencing emotions fully and being open to internal experiences without trying to change or avoid them (Hayes et al., 1999; Wolgast et al., 2011). In laboratory studies, both strategies have been shown to alleviate subjective negative emotions and adverse physiological effects (Hofmann et al., 2009; Wolgast et al., 2011; Keng et al., 2013; Troy et al., 2018).

Previous studies have demonstrated that reappraisal recruits cognitive control regions, such as the prefrontal cortex and inferior parietal lobule (Ochsner et al., 2012; Buhle et al., 2014; Frank et al., 2014; Morawetz et al., 2017) and involves the negative functional coupling between the prefrontal cortex and amygdala (Sarkheil et al., 2019; Berboth and Morawetz, 2021). Contrastingly, brain activity during acceptance has begun to be examined, but its functional connectivity has been less explored. Previous studies have found that acceptance decreases activation in the precuneus and posterior cingulate cortex, suggesting that acceptance reduces self-reflection (Dixon et al., 2020; Messina et al., 2021), which is a process of reflecting on one’s own characteristics, abilities, and attitudes (Johnson et al., 2002). Additionally, Goldin et al. (2019) found that acceptance recruited less activation of cognitive control regions compared with that of reappraisal. Regarding functional connectivity, Kober et al. (2019) investigated the functional coupling of acceptance of pain and unpleasant images but did not find statistically significant functional coupling with the amygdala or insula compared with the natural reaction. To date, no study has compared the functional connectivity of acceptance with that of other adaptive strategies, such as reappraisal, which may help clarify the unique neural mechanisms of acceptance compared with others by focusing on differences between emotion regulation strategies.

Brain activity has been suggested to vary with the complexity of stimulus materials and the type of emotions (Kim and Hamann, 2007; Vytal and Hamann, 2010; Dörfel et al., 2014; Saarimäki et al., 2016; Tsujimoto et al., 2022). Several studies have investigated the neural bases of regulating emotions induced by personal events and discrete emotions (Fabiansson et al., 2012; Zeidan et al., 2014; Goldin et al., 2019). Increased activity of fear circuits, such as the amygdala and insula, has been found in anticipatory anxiety, and reappraisal in anticipatory anxiety was associated with increased activity in prefrontal regions and decreased amygdala activation (Yoshimura et al., 2014). Additionally, reappraisal in social anxiety autobiographical scripts was related to the negative functional coupling between the left amygdala and bilateral prefrontal cortex (Goldin et al., 2009). Furthermore, acceptance was associated with greater activation in the anterior cingulate cortex than thinking about worries in individuals with generalized anxiety disorder (Ellard et al., 2017). However, the neural basis of acceptance of anxiety in healthy adults remains unclear, and no study has compared the neural bases of reappraisal and acceptance for individuals’ anxious events. Identifying neural mechanisms of emotion regulation that focus on discrete emotions and materials can contribute to a further understanding of emotion regulation processes.

The present study investigated the brain activation and functional connectivity of reappraisal and acceptance for anxious events. We hypothesized that reappraisal would be associated with higher activation of the cognitive control regions than other strategies, whereas acceptance would deactivate the posterior cingulate cortex and precuneus. Regarding functional connectivity, we used the region of interest (ROI) of the amygdala and ventral anterior insula. The anterior insula is related to anxiety and interoception (Paulus and Stein, 2006; Terasawa et al., 2013), and particularly, the ventral anterior insula is strongly involved in emotional awareness and the experience of anxiety (Carlson et al., 2011; Denny et al., 2014). We expected that reappraisal would show stronger negative connectivity of cognitive control regions and anxiety-processing regions than thinking about anxious events. Finally, we hypothesized that acceptance would have stronger functional connectivity between the regions related to self-reflection and anxiety-processing than other strategies.

## Materials and Methods

### Participants

Human participants were recruited at Tohoku University, Japan. Forty-one healthy college students participated in an fMRI study of emotion regulation and executive control (this paper reports only the data on emotion regulation). According to the Edinburgh Handedness Inventory – Short Form (Veale, 2014), all participants were right-handed (mean: 92.44 ± 11.59). Participants who presently had or reported pregnancy, claustrophobia, history of mental disorders, metallic implants in the body, or medication use that affects cognitive function were excluded. In addition, we excluded the data from three participants who fell asleep during the task, two participants whose images were not transferred successfully from the MRI console to the server, and one participant with an average framewise displacement (Power et al., 2012) >0.3 mm. Therefore, the data from 35 participants (13 females, mean age: 20.6 ± 1.8, range: 18–25 years) were analyzed. The experiment was conducted in accordance with the Declaration of Helsinki, and all procedures were approved by the Institutional Review Board of the Smart-Ageing Research Center of Tohoku University.

### Procedure

The individuals participated in this experiment for three consecutive days. They reported keywords of three unresolved anxiety-inducing situations (Keng et al., 2013; Shields et al., 2016), which were used as stimuli for emotion regulation tasks involving reappraisal, acceptance, and the control conditions ∼3 d before participating in the experiment. On the first day, the participants were briefed about the experiment, provided informed consent, received instructions and examples of an emotion regulation strategy, and practiced the tasks under one of the three conditions. The instructions were developed based on previous studies (Keng et al., 2013; Noguchi et al., 2017). Reappraisal involved changing one’s thoughts and interpretations of anxiety-inducing situations. Acceptance meant accepting one’s emotions, thoughts, and body sensations, even if they were uncomfortable, without trying to change, control, or avoid them. In the control condition, participants thought about their unresolved anxiety-inducing situations. The emotion regulation task was practiced during two trials. After practicing, they worked on the first cognitive task, the emotion regulation task with the instructed strategy, and the second cognitive task in an MRI scanner. After the scan, they were asked to rate how well they could implement the strategy during an emotion regulation task (1, not at all; 7, very successfully) outside the scanner. This value was used to indicate whether the emotion regulation strategies could be applied rather than whether anxiety was reduced. On the second and third days, the participants practiced and performed the tasks in the same manner as that on the first day. For the emotion regulation task, the participants used a different emotion regulation strategy than on the previous day. On the second day, they answered several questionnaires after completing the tasks (not reported in this article). The participants received JPY 3000 for their participation in the study.

### Emotion regulation task

**Figure 1.**
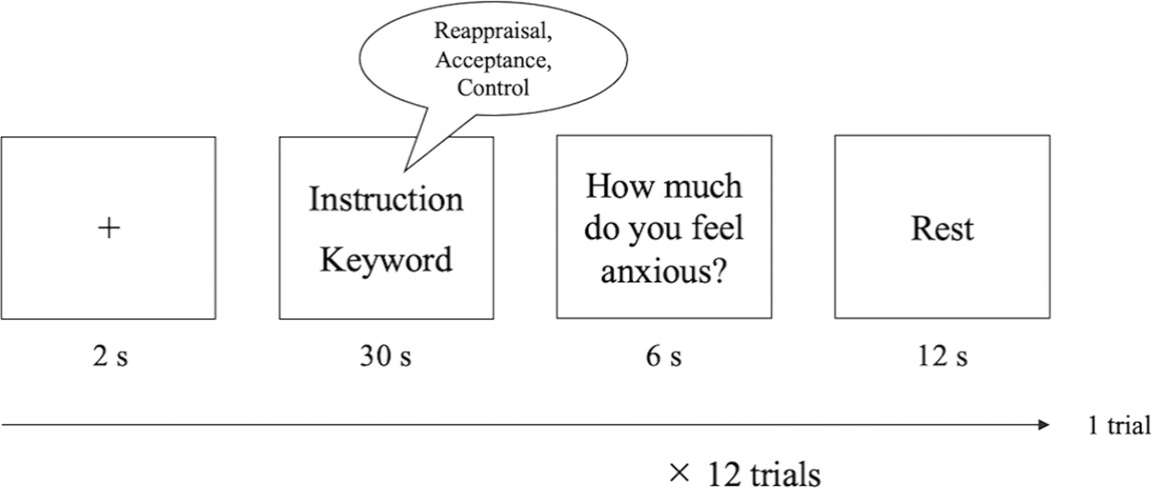
Schematic of one trial. Each trial included a fixation cross for 2 s, an emotion regulation phase for 30 s, a rating phase for 6 s, and a rest phase for 12 s.

We used reappraisal, acceptance, and control as the three conditions. Each day’s emotion regulation task consisted of 12 trials in one of the three conditions. During each trial, a fixation cross was presented for 2 s, followed by 30 s of an instructed strategy and an emotion-inducing keyword, during which participants engaged in emotion regulation. The keywords were those reported by the participants in advance (described above, Procedure), and each keyword was displayed four times on each day in a random order. The participants then rated the degree of anxiety they felt at that moment on a seven-point Likert scale, with 1 representing “not at all” and 7 representing “very much” by pressing one of seven response keys. At the end of the trial, a “rest” message was displayed, and the participants were instructed to rest for 12 s (Fig. 1).

### MRI data acquisition and imaging parameters

Image acquisition was performed with a 3 T MRI scanner (Philips Achieva dStream 3.0T). fMRI data were acquired with T2-weighted gradient echo-planar imaging. Specifically, 32 slices of gradient-echo images (echo time = 30 ms, flip angle = 80°, thickness = 3 mm, field of view = 192 mm, matrix = 64 × 64, slice gap = 0.5 mm, and voxel size = 3 × 3 × 3 mm) were acquired with a repetition time of 2000 ms. The T1-weighted anatomic images (thickness = 1 mm, field of view = 240 mm, matrix = 240 × 240) were acquired using magnetization-prepared, rapid-acquisition gradient-echo at the end of the third experimental day.

### Analysis

#### Behavioral data analysis

To evaluate whether the participants were able to use the strategy, the successful implementation of reappraisal and acceptance was compared using a *t* test. One-way repeated measures analysis of variance was used in anovakun version 4.8.5 to analyze differences in anxiety reduction of the cognitive emotion regulation conditions (reappraisal and acceptance) and the control condition. Independent variables were the conditions, and dependent variables were the mean of anxiety ratings for each condition per participant. Multiple comparisons were performed by Shaffer’s modified sequential rejection Bonferroni procedure. All behavioral data analyses were performed using R version 4.0.0 (R Core Team; https://www.R-project.org/).

#### fMRI data preprocessing

Image preprocessing was performed using SPM 12 (http://www.fil.ion.ucl.ac.uk/spm/software/spm12/) and MATLAB R2020b (MathWorks). Preprocessing included correction for realignment, slice timing correction, co-registration, segmentation of structural images, spatial normalization using the anatomic image and the Montreal Neurologic Institute template, and smoothing using a Gaussian kernel with full-width at a half-maximum of 8 mm.

#### Whole-brain analysis

Statistical fMRI analyses were performed using SPM12. A general linear model was specified for each participant to model the BOLD response using a canonical hemodynamic response function at the individual level. The duration of emotion regulation (30 s) and six movement parameters were entered as regression variables. At the group-level, paired *t* tests were conducted with contrasts: reappraisal > control, acceptance > control, and reappraisal > acceptance, as well as the inverse contrasts. The results of the group-level analysis were assessed at a threshold of *p *<* *0.001 at the voxel level and *p *<* *0.05 with false discovery rate (FDR) correction at the cluster level. Regions were labeled using the SPM Anatomy Toolbox (Eickhoff et al., 2005).

#### Functional connectivity analysis

Functional connectivity analysis was performed using the CONN toolbox, version 20.b (www.nitrc.org/projects/conn, RRID: SCR_009550; Whitfield-Gabrieli and Nieto-Castanon, 2012) to investigate how the time course of the anxiety processing regions, such as the amygdala (Ellard et al., 2017; Babaev et al., 2018) and ventral anterior insula (Paulus and Stein, 2006; Carlson et al., 2011; Denny et al., 2014) related to other brain regions. The ROI of the left and right amygdala was defined using the Wake Forest University PickAtlas (https://www.nitrc.org/projects/wfu_pickatlas/; Maldjian et al., 2003) with the automatic anatomic labeling atlas (Tzourio-Mazoyer et al., 2002). Additionally, we obtained the predefined ROI of the left and right ventral anterior insula directly from a previous study (Deen et al., 2011), in which the insular lobe was divided into three subregions by clustering of functional connectivity patterns. The functional data preprocessing consisted of realignment and unwrapping, slice timing correction, outlier detection with ART, segmentation and normalization, and smoothing. Segmentation and normalization were used as preprocessing for the structural data. Parameters for realignment and scrubbing were entered as first-level covariates. MRI data for 30 s × 12 trials of emotion regulation were used for analysis. ROI-to-voxel analyses for each participant were conducted with the ROI of the left and right amygdala and ventral anterior insula. At the group level, paired *t* tests were conducted to compare the functional connectivity of the anxiety processing regions with other regions among the conditions (reappraisal > control, acceptance > control, reappraisal > acceptance, and the inverse relationships). The results were evaluated at a threshold of *p *<* *0.001 at the voxel level and *p *<* *0.05 at the cluster level with FDR correction.

### Data and code accessibility

The data and code described in the paper are freely available online at https://osf.io/nyrws/. The code is available as Extended Data 1.

## Results

### Behavioral results

To confirm whether the participants were able to use the strategy, the degree of perceived success of emotion regulation for each participant was collected outside the MRI scanner immediately after the task was completed. No statistically significant difference was observed between the successful implementation of reappraisal and that of acceptance [reappraisal: 5.26 ± 1.31, acceptance: 5.31 ± 1.23, *t*_(34)_ = −0.18, *p *=* *0.86, 95% confidence interval (CI) [−0.691–0.577], Cohen’s *d* = −0.04].

**Figure 2.**
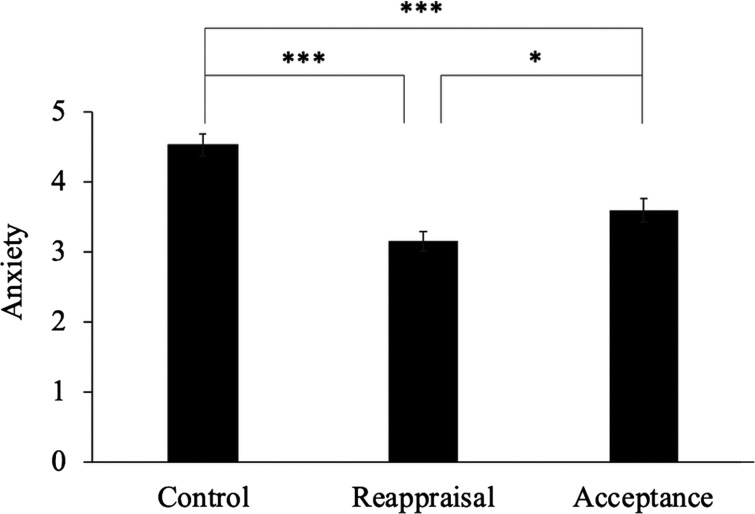
Subjective rating of anxiety. ***p <* *0.001, *p <* *0.05. The *p*-values were corrected by Shaffer’s modified sequential rejection Bonferroni procedure. Error bars = SEM.

We compared the effects of reappraisal, acceptance, and the control condition on reducing anxiety. There was a statistically significant main effect of the conditions (*F*_(2,68)_ = 31.18, *p *<* *0.001, η^2^ = 0.28). The *post hoc t* tests indicated a greater reduction in anxiety for reappraisal (mean = 3.15 ± 0.84, *t*_(34)_ = 7.86, *p *<* *0.001, 95% CI [−1.622–−1.121], *d* = −1.56) and acceptance (mean = 3.59 ± 1.01, *t*_(34)_ = 4.92, *p *<* *0.001, 95% CI [−1.205–−0.661], *d* = −0.97) than for the control condition (mean = 4.53 ± 0.91; Fig. 2). Compared with acceptance, reappraisal was more effective in reducing anxiety (*t*_(34)_ = 2.61, *p *<* *0.05, 95% CI [−0.680–−0.198], *d* = −0.47).

### Brain activation

**Figure 3.**
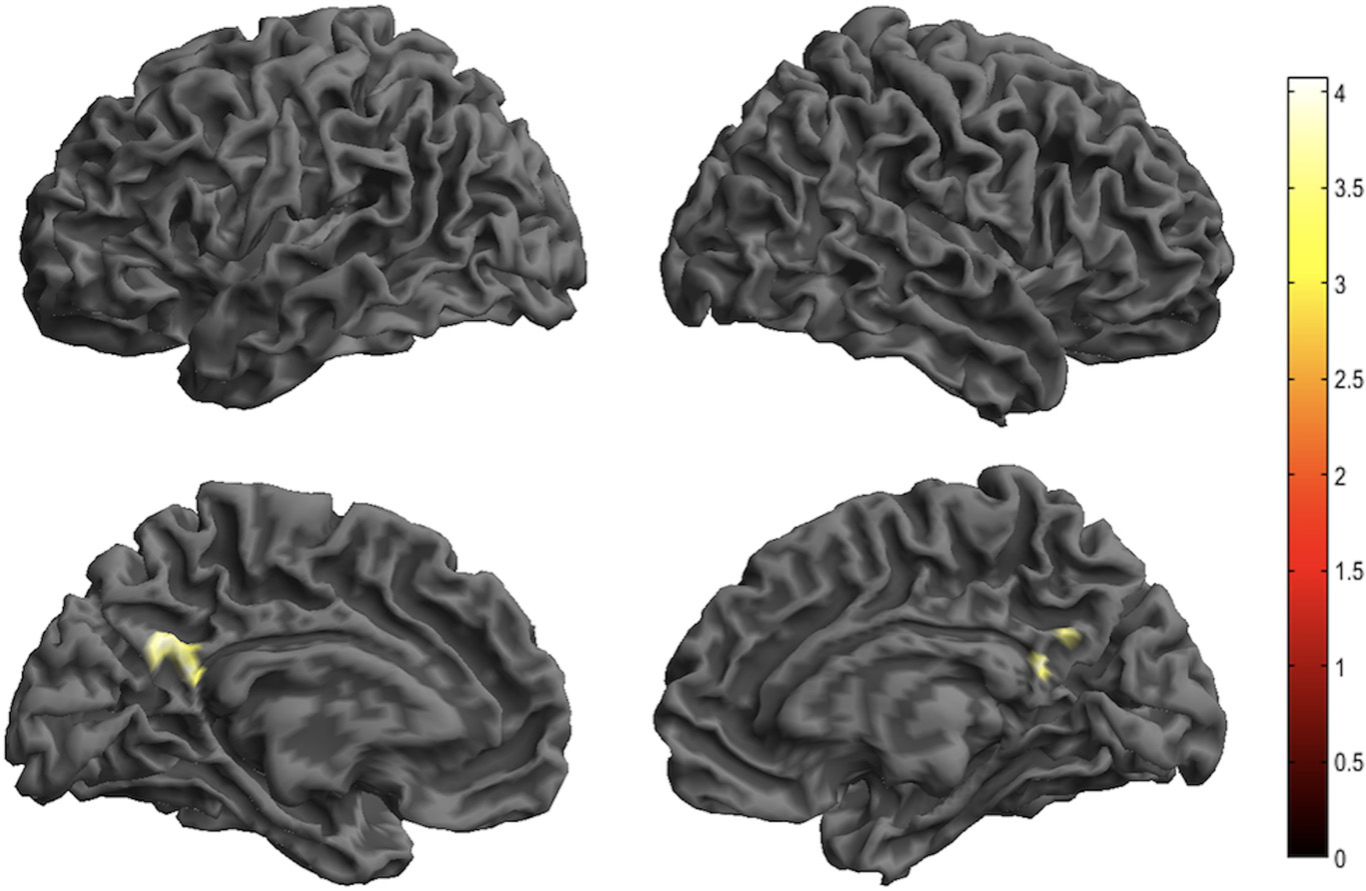
Whole-brain analysis for reappraisal > acceptance. Threshold: *p *<* *0.001 uncorrected at the voxel level and *p *<* *0.05 with FDR correction at the cluster level. FDR = false discovery rate.

Table 1 shows the results of the comparison of brain activation under various conditions. No voxels with significantly increased or decreased activity survived in reappraisal or acceptance compared with the control condition. Reappraisal increased activation in the bilateral posterior cingulate cortex and precuneus significantly more than acceptance (Fig. 3; Table 1). No significantly increased activation was observed during acceptance compared with reappraisal.

**Table 1:** Results of whole-brain analysis

Contrast		Coordinates			Peak		Cluster
hemisphere	Region	*x*	*y*	*z*	T	k	p(FDR)
Reappraisal > acceptance							
L	Posterior cingulate gyrus	−4	−52	18	4.35	350	0.027

FDR = false discovery rate.

Contrasts that were not statistically significant were omitted. *p *<* *0.001 (uncorrected) at the voxel level and *p *<* *0.05 with FDR correction at the cluster level.

### Functional connectivity

**Figure 4.**
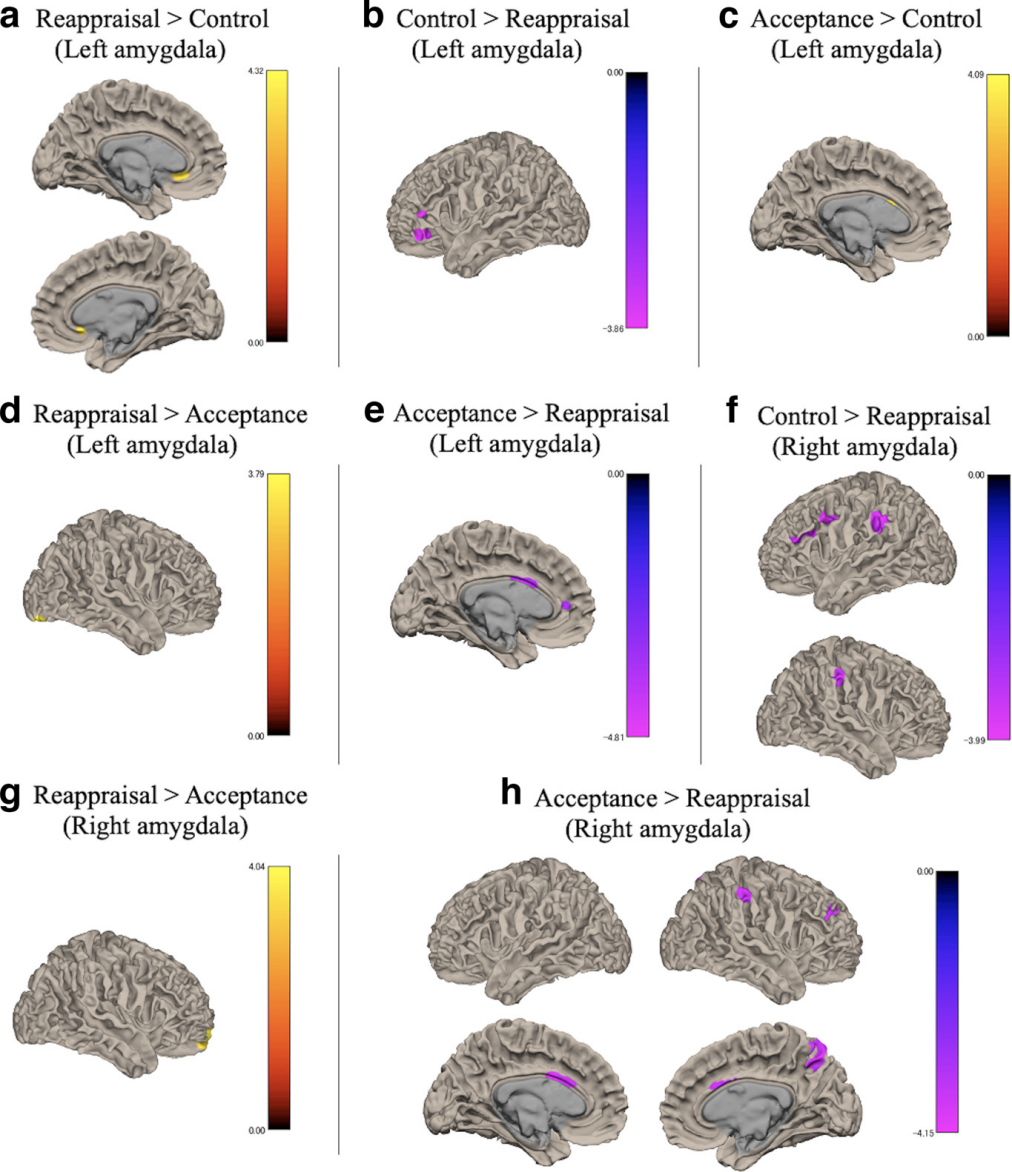
Functional connectivity between the left amygdala and other brain regions for (***a***) reappraisal > control, (***b***) control > reappraisal, (***c***) acceptance > control, (***d***) reappraisal > acceptance, (***e***) acceptance > reappraisal, and between the right amygdala and other brain regions for (***f***) control > reappraisal, (***g***) reappraisal > acceptance, (***h***) acceptance > reappraisal. Threshold: *p *<* *0.001 uncorrected at the voxel level and *p *<* *0.05 with FDR correction at the cluster level. FDR = false discovery rate.

**Figure 5.**
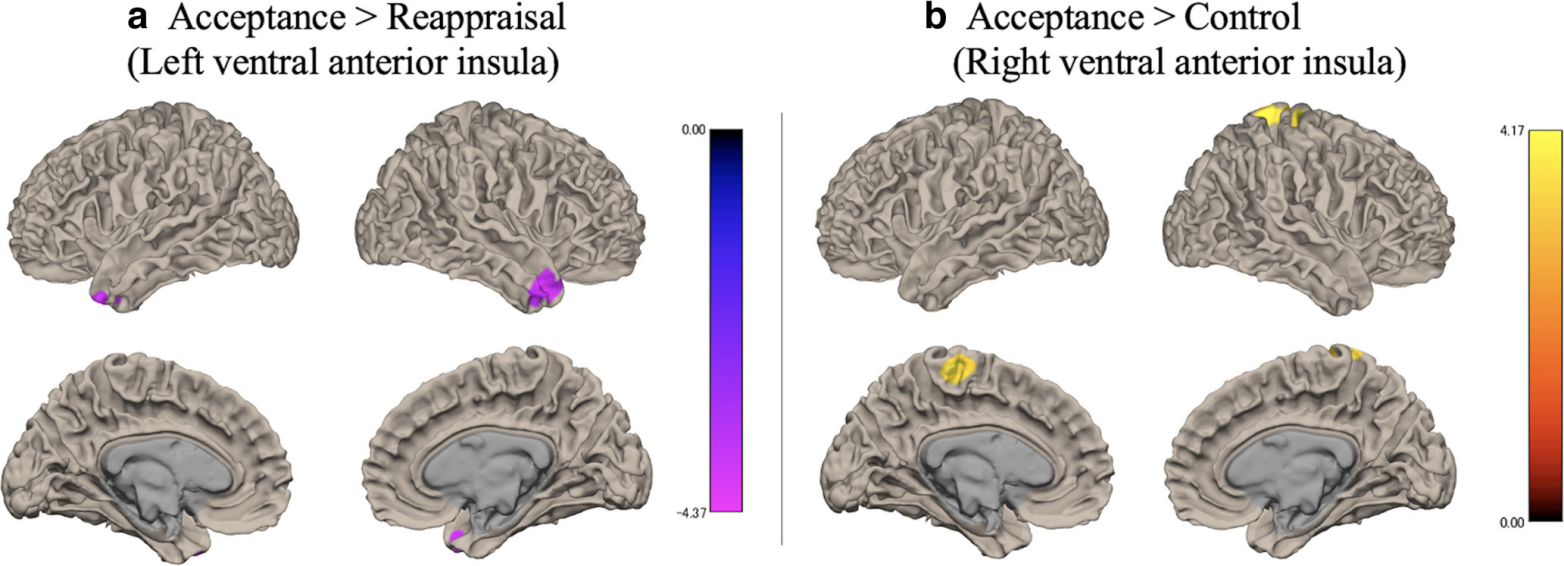
Functional connectivity between the left ventral anterior insula and other brain regions for (***a***) acceptance > reappraisal and between the right ventral anterior insula and other brain regions for (***b***) acceptance > control. Threshold: *p *<* *0.001 uncorrected at the voxel level and *p *<* *0.05 with FDR correction at the cluster level. FDR = false discovery rate.

Figures 4 and 5 show the results of the functional connectivity analysis.

#### Functional connectivity with the amygdala

Reappraisal showed significantly positive correlations with the left amygdala in the bilateral anterior cingulate cortex, left thalamus proper, hippocampus, and middle cingulate cortex compared with the control condition (Table 2). Significantly more negative correlations were found between the left amygdala and the left inferior frontal gyrus during reappraisal compared with the control condition. During acceptance, significantly more positive correlations were observed between the left amygdala and left caudate compared with the control. However, no significant negative correlations were observed in acceptance compared with the control condition.

**Table 2:** Results of functional connectivity analysis of the left amygdala

Contrast			Coordinates			Peak		Cluster
correlation	Hemisphere	Region	*x*	*y*	*z*	T	k	p(FDR)
Reappraisal > control								
Positive correlations	L	Anterior cingulate gyrus	−6	26	−2	5.13	117	0.009
	L	Thalamus proper	−16	−34	14	4.71	158	0.004
	L	Middle cingulate gyrus	−18	−16	34	4.44	69	0.040
Negative correlations	L	Inferior frontal gyrus	−30	38	6	4.69	157	0.008
Acceptance > control								
Positive correlations	L	Caudate	−18	−16	30	5.70	302	0.000
	L	Caudate	−6	16	16	5.33	152	0.002
Negative correlations	None							
Reappraisal > acceptance								
Positive correlations	R	Fusiform gyrus	26	−86	−16	4.30	114	0.016
Negative correlations	R	Thalamus proper	4	−20	−2	5.46	73	0.046
	L	Middle cingulate gyrus	−8	10	26	5.11	148	0.008
	L	Anterior cingulate gyrus	−22	46	4	4.31	126	0.009

FDR = false discovery rate.

*p *<* *0.001 (uncorrected) at the voxel level and *p *<* *0.05 with FDR correction at the cluster level.

Compared with acceptance, reappraisal exhibited significantly positive functional connectivity in the right fusiform gyrus and negative functional connectivity in the bilateral thalamus proper, left anterior and middle cingulate cortex with the left amygdala.

Additionally, significant negative correlations were observed between the right amygdala and bilateral supramarginal gyrus, left precentral gyrus, and middle and inferior frontal gyrus during reappraisal compared with the control condition (Table 3). We did not find significant functional coupling with the inverse contrasts. Moreover, acceptance did not show significant functional connectivity in any regions with the right amygdala compared with the control condition.

**Table 3.** Results of functional connectivity analysis of the right amygdala

Contrast			Coordinates			Peak		Cluster
correlation	Hemisphere	Region	*x*	*y*	*z*	T	k	p(FDR)
Reappraisal > control								
Positive correlations	None							
Negative correlations	L	Precentral gyrus	−46	6	34	4.54	139	0.016
	L	Supramarginal gyrus	−66	−36	40	4.12	119	0.016
	L	Middle frontal gyrus	−42	22	22	3.85	82	0.042
	R	Supramarginal gyrus	60	−30	46	3.76	76	0.042
Reappraisal > acceptance								
Positive correlations	R	Frontal pole	12	70	−12	4.80	156	0.004
Negative correlations	R	Middle frontal gyrus	30	34	24	5.05	96	0.026
	L	Middle cingulate gyrus	−4	4	28	4.28	123	0.023
	R	Supramarginal gyrus	58	−30	50	4.22	84	0.031
	R	Precuneus	6	−64	50	4.15	110	0.023

FDR = false discovery rate.

Contrasts that were not statistically significant were omitted. *p *<* *0.001 (uncorrected) at the voxel level and *p *<* *0.05 with FDR correction at the cluster level.

During reappraisal compared with acceptance, significant positive correlations were observed between the right amygdala and right frontal pole and medial orbital gyrus. Additionally, reappraisal showed negative functional connectivity between the right amygdala and right precuneus, supramarginal gyrus, middle frontal gyrus, and bilateral anterior and middle cingulate cortex.

#### Functional connectivity with the ventral anterior insula

Negative functional coupling with the left ventral anterior insula emerged in the bilateral temporal pole in reappraisal compared with acceptance (Table 4). However, no significant positive correlations were observed in the inverse contrasts. Additionally, compared with the control condition, reappraisal and acceptance did not show significant functional coupling with the left ventral anterior insula.

**Table 4.** Results of functional connectivity analysis of the left ventral anterior insula

Contrast			Coordinates			Peak		Cluster
correlation	Hemisphere	Region	*x*	*y*	*z*	T	k	p(FDR)
Reappraisal > acceptance								
Positive correlations	None							
Negative correlations	R	Temporal pole	52	8	−30	5.34	252	0.000
	L	Temporal pole	−40	14	−46	4.77	91	0.045

FDR = false discovery rate.

Contrasts that were not statistically significant were omitted. *p *<* *0.001 (uncorrected) at the voxel level and *p *<* *0.05 with FDR correction at the cluster level.

Compared with the control condition, acceptance showed significant positive correlations between the right ventral anterior insula and bilateral precentral gyrus and right postcentral gyrus (Table 5). No significant negative functional coupling with the right ventral anterior insula was observed in acceptance compared with the control. Finally, no significant functional connectivity with the right ventral anterior insula was observed in reappraisal compared with the other conditions.

**Table 5.** Results of functional connectivity analysis of the right ventral anterior insula

Contrast			Coordinates			Peak		Cluster
correlation	Hemisphere	Region	*x*	*y*	*z*	T	k	p(FDR)
Acceptance > control								
Positive correlations	R	Postcentral gyrus	20	−40	72	4.69	162	0.009
	L	Precentral gyrus	−2	−26	64	4.36	112	0.024
Negative correlations	None							

FDR = false discovery rate.

Contrasts that were not statistically significant were omitted. *p *<* *0.001 (uncorrected) at the voxel level and *p *<* *0.05 with FDR correction at the cluster level.

## Discussion

The present study is the first to directly compare the neural bases of reappraisal and acceptance for anxious events. Reappraisal was the most effective way to reduce anxiety, which is consistent with the findings of a previous study (Hofmann et al., 2009). Contrary to expectations, no statistically significant differences were observed in the brain activation levels between cognitive emotion regulation strategies and the control condition. However, acceptance decreased activation in the posterior cingulate cortex and precuneus more than reappraisal. Moreover, functional coupling with the amygdala and ventral anterior insula distinguished the emotion regulation strategies for anxiety. Reappraisal showed strong negative functional connectivity between the amygdala and cognitive control regions, such as the middle and inferior frontal gyrus and supramarginal gyrus.

With respect to behavioral data, reappraisal and acceptance significantly decreased anxiety compared with the control condition. This result is consistent with previous research showing the effectiveness of these emotion regulation strategies in decreasing negative emotions (Hofmann et al., 2009; Wolgast et al., 2011; Troy et al., 2018; Goldin et al., 2019) and indicates that reappraisal and acceptance are also effective for an individual’s anxious events.

Contrary to the hypothesis that levels of activation in the regions would differ depending on the emotion regulation strategies, no significant difference was observed in brain activity levels of reappraisal and acceptance compared with those of the control condition. Our results reflect the possibility that brain activity does not differ between continuing to think about emotion-inducing personal events and cognitive emotion regulation strategies. This possibility was partially supported by a study comparing brain activity during reappraisal and rumination in personal anger, which found no difference in brain activity levels (Fabiansson et al., 2012).

Reappraisal showed greater activation of the bilateral posterior cingulate cortex and precuneus, which are related to self-reflective processes (Cavanna and Trimble, 2006; Cavanna, 2007; Brewer et al., 2013) than acceptance. Therefore, reappraisal may strengthen self-relevant information, whereas acceptance may weaken self-reflective information. Additionally, a previous study suggested that increased posterior cingulate cortex activity is involved in effortful cognitive control (Garrison et al., 2013), and our results may reflect the differences in effortful cognitive load manifested in the differences in brain activity during reappraisal and acceptance.

Consistent with our hypothesis, differences in functional connectivity with the amygdala were observed between reappraisal and the control condition. Reappraisal exhibited stronger negative correlations between the right amygdala and the middle and inferior frontal gyrus and supramarginal gyrus compared with the control. The amygdala is related to the evaluation of anxiety (Ellard et al., 2017; Babaev et al., 2018), and the right amygdala, in particular, is associated with fast and automatic responses to stimuli (Costafreda et al., 2008). The middle and inferior frontal gyrus and supramarginal gyrus play important roles in cognitive control, such as selective attention, working memory, and inhibition (Ochsner et al., 2012; Barbey et al., 2013). Moreover, our results are consistent with the findings of a previous meta-analysis (Berboth and Morawetz, 2021), and suggest that on reappraisal, the cognitive control areas regulate amygdala activity to achieve reduction of personal anxiety, especially modulation of the initial emotional changes caused by personal anxious events.

Furthermore, reappraisal showed positive functional connectivity between the left amygdala and the subgenual anterior cingulate cortex, in addition to negative functional coupling between the left amygdala and the inferior frontal gyrus compared with the control condition. The left amygdala is engaged in language or delayed, evaluative responses (Costafreda et al., 2008). Prior studies have shown reduced activity in the amygdala and subgenual anterior cingulate cortex because of emotion regulation (Kanske et al., 2011), and these regions were co-activated during the perception of negative stimuli (Pezawas et al., 2005). Thus, reappraisal may alter the broader emotion-processing system.

In contrast, acceptance showed positive functional connectivity between the left amygdala and the caudate compared with the control condition. The caudate is involved in the perception of emotional information (Delgado et al., 2004; Kemp et al., 2013), suggesting that acceptance may alter the perception of anxiety. Furthermore, reappraisal showed negative functional coupling between the amygdala and middle frontal gyrus, supramarginal gyrus, precuneus, and anterior and middle cingulate cortex compared with acceptance. Thus, the involvement of self-reflective processes may differ between reappraisal and acceptance, and reappraisal controls amygdala activity more through cognitive control compared with acceptance.

Acceptance showed stronger positive functional coupling between the ventral anterior insula and precentral gyrus and postcentral gyrus compared with the control condition. The ventral anterior insula is involved in anxiety, interoceptive sense, and emotional anticipation (Paulus and Stein, 2006; Berntson et al., 2011; Carlson et al., 2011; Alvarez et al., 2015). The postcentral gyrus, together with the anterior insula, is related to interoceptive sense (Craig, 2002; Min et al., 2022); and the precentral gyrus is known to be involved in self-image (Théoret et al., 2004) and voluntary motor control (Banker and Tadi, 2019). Therefore, in acceptance, the emotional anticipation and self-reflective processes are likely synchronized. In contrast, reappraisal had negative functional coupling between the ventral anterior insula and the temporal pole compared with acceptance. The temporal pole is implicated in a variety of functions, including emotional and social behavior and semantic processing. In particular, this region has been suggested to play a role in linking complex processed perceptual inputs to visceral emotional responses (Olson et al., 2007; Córcoles-Parada et al., 2019). Thus, our results may reflect differences in the complexity of emotional processes between reappraisal and acceptance.

This study had several limitations. First, because this study limited the target population to college students, further investigation in different age groups is needed to generalize the results (Allard and Kensinger, 2014; Mather, 2016). Second, the proportion of female participants in this study was low. Third, we did not use physiological measures for anxious reactions. The use of physiological measures would allow us to objectively assess the effectiveness of anxiety regulation. Finally, in this study, the cognitive task was performed before the emotion regulation task, which may have affected brain activity during emotion regulation despite the control between conditions.

Nevertheless, this study contributes to a better understanding of emotion regulation processes by providing information on brain activity and functional connectivity in reappraisal and acceptance for personal anxious events. Reappraisal strengthened the negative functional coupling between the cognitive control areas and emotion-processing regions, while acceptance was characterized by a reduction in the self-reflection process. Our findings will help determine interventions aimed at mitigating the negative effects of anxiety.

10.1523/ENEURO.0033-23.2023.ext1 Extended Data 1 Scripts for the analyses used in the paper. Download Extended Data 1, ZIP file.

## Synthesis

Reviewing Editor: Alexander Soutschek, Ludwig-Maximilians-Universitat Munchen Decisions are customarily a result of the Reviewing Editor and the peer reviewers coming together and discussing their recommendations until a consensus is reached. When revisions are invited, a fact-based synthesis statement explaining their decision and outlining what is needed to prepare a revision will be listed below. The following reviewer(s) agreed to reveal their identity: NONE.

1) The relationship between the aim of the study (“In light of the negative effects described above, this functional MRI (fMRI) study examined the neural basis of emotion regulation strategies for anxiety induced by the participants’ personal events”) and the negative effects of anxiety described in the first lines of the introduction is not clear. If the aim of the study is solely descriptive (neural basis of emotion regulation strategies), this has to be clarified.

In other words, the question is whether the present study helps reducing/managing anxiety negative effects in some ways? If yes, please specify how.

2) It remains quite obscure what is actually missing in the literature regarding acceptance (lines 66-69). Please specify the gap in the literature, referring to the meta-analysis previously cited (line 62, Messina et al.).
